# Paracetamol Versus Ondansetron for Prevention of Postoperative Shivering in Liposuction Surgeries Under Combined General Epidural Anaesthesia: A Randomized Controlled Trial

**DOI:** 10.4274/TJAR.2022.22927

**Published:** 2023-06-16

**Authors:** Amr Samir Wahdan, George Eshak Loza, Hussain Othman Alshehri, Ahmed Farag Shedid, Atef Kamel Salama, Wessam Samir Wahdan, Mennatallah Magdi Mohamed

**Affiliations:** 1 Department of Anaesthesia, Surgical ICU and Pain Management, Cairo University Faculty of Medicine, Cairo, Egypt; 2 Department of Anaesthesia, Al-hada Armed Force Hospital, Taif, Saudi Arabia; 3 Department of Plastic and Reconstructive Surgery, Cairo University Faculty of Medicine, Cairo, Egypt

**Keywords:** Lipectomy, ondansetron, paracetamol, postoperative complications, shivering

## Abstract

**Objective:**

Postoperative shivering (POS) is considered one of the most common complications that is encountered by the anaesthetists worldwide. Despite using several treatment options, there has not been a clear consensus regarding this issue. This trial was conducted to investigate the efficacy and safety of paracetamol and ondansetron in preventing POS in patients undergoing liposuction procedures under combined general epidural anaesthesia.

**Methods:**

One hundred twenty patients scheduled for liposuction were randomly allocated to one of three groups: group P (paracetamol group) which received 1 g paracetamol intravenously, group O (ondansetron group) which received 8 mg of ondansetron intravenously, and group S (saline group), which received 100 mL normal saline intravenously; all medications were given postoperatively. The primary outcome was the incidence of POS, and the secondary outcomes included shivering score, tympanic temperature, and the occurrence of side effects.

**Results:**

The incidence of occurrence of POS was found to be lower in groups P and O compared to group S with values of 25% and 37.50% vs. 77.50%, respectively, with a *P* value <0.001. Additionally, the severity of POS was found to be lower in groups P and O compared to group S (*P* <0.001). Tympanic temperature and complications were comparable between the groups with no significant differences.

**Conclusion:**

Prophylactic use of paracetamol or ondansetron at the end of the procedure was shown to be of great value in reducing the incidence and severity of POS, with no statistically significant difference between the paracetamol and ondansetron groups. Moreover, no significant drawbacks were reported as a result of using these medications.

**Main Points**

• One of the most common postoperative complications is shivering in the recovery room. Many treatment options have been used in management of this adverse effect, but there is no consensus.

• Paracetamol is as effective as ondansetron when administrated at the end of surgery and can reduce the incidence and severity of postoperative shivering.

## Introduction

Postoperative shivering (POS) is defined as detectable oscillations in skeletal muscle that are frequent, spontaneous, involuntary, and asynchronous, starting 5-30 min after induction of anaesthesia causing noticeable increase in the core temperature. It is mainly caused because of hypothermia that occurs following the use of general or regional anaesthesia techniques. However, POS was also detected in normothermic patients because of pyrogenic agent release postoperatively.^1,2^

POS is considered one of the commonest complications that occurs as a result of using general anaesthesia,^3^ with an incidence varying between 5%-65% in patients who receive general anaesthesia and 30%-55% in those who receive regional anaesthesia. It is usually related to several risk factors including the patients’ age, operating room temperature, gender and the operative time.^4^ In addition to the discomfort caused by POS; it also causes an increase in both oxygen consumption and carbon dioxide production, thus resulting in hypoxemia and an increase in the lactic acid level. Moreover, it increases the sympathetic outflow because of catecholamine release, which can aggravate ischemic cardiac conditions in known cardiac patients as well as increase intracranial pressure and intraocular pressure.^5^

Therefore, preventing POS will not only reduce an unpleasant side effect of anaesthesia but also prevent postoperative complications.^6^ To prevent POS, several techniques were used to prevent hypothermia, e.g., increasing the operating room temperature, using warm fluid infusion and forced air warmers, or administering pharmacological agents. It is always preferable to prevent post-anaesthesia shivering rather than to treat it once it develops.^7,8^

A wide diversity of pharmacological agents has been used to prevent or treat POS, including opioids (pethidine and tramadol) paracetamol, dexmedetomidine, ondansetron, ketamine, dexamethasone, and ephedrine. However, most of these pharmacological agents have undesirable side effects which render them unsuitable for use as anti-shivering agents.^9,10,11,12^

The goal of our trial was to measure the efficacy and safety of using paracetamol or ondansetron in reducing the incidence and severity of POS in patients undergoing mega liposuction procedures under a combined general epidural anaesthesia technique.

## Methods

This prospective randomized controlled trial was conducted in general surgery operating rooms between January 2021 and January 2022. The approval of the Institute Ethical Committee was obtained [N-122-2020]. Informed written consent was signed by each participant before enrollment in this trial. One hundred twenty subjects aged between 18 and 40 years with an ASA physical score I-III, scheduled for elective liposuction surgery under combined epidural and general anaesthesia were enrolled in this study. Participants were excluded from this study if they refused to participate, had a body mass index >50 kg m2^-1^ or ASA higher than III, had a history of any of the following co-morbidities: renal, hepatic, or thyroid disorders or seizures, allergy to the study drugs, and abnormal body temperature (less than or 36.5°C or more than 37.5°C). The same anaesthetic and surgery teams were involved in the procedure for all patients.

One day before the procedure, all participants were scheduled for preoperative evaluation in the form of medical history, physical examination, and routine laboratory values including complete blood count, coagulation profile, AST, ALT, urea, and creatinine. A full explanation of the study protocol was provided to the patients, including the drugs used and the anaesthesia technique. They were also informed that they could discontinue participation in the study whenever they desired.

Two anaesthesiologists were involved in the research, one who performed the randomization process and another who recorded the data. The study drug was transferred from its original vial to be prepared and labeled with the patient identification number in a specific Burette Set (Dosifix^®^, B. Braun). The study solution contained 1,000 mg of acetaminophen with a volume of 100 mL or 8 mg of ondansetron in 100 mL of normal saline or 100 mL of normal saline (for control group). A pharmacist not involved in conducting the trial; prepared the drug based on a randomization table, taking all precaution measures to guaranty the blinding of the anaesthesiologists and surgeons. Fortunately, both paracetamol and ondansetron are clear solutions, thus undistinguishable from the placebo saline solution.

On the day of the procedure, after confirming sufficient fasting time, the patients were taken to the preoperative-holding area. Demographic data were reported, an intravenous line was inserted using a 20-G intravenous (IV) cannula, and intravenous 0.01 mg kg^-1^ of midazolam, 0.15 mg kg^-1^ of metoclopramide, 8 mg of dexamethasone, and 40 mg of pantoprazole were. They were then transferred to the operating room; the operating room temperature was adjusted between 22°C-24°C.

Upon arrival to the OR, the standard routine monitors were attached to the patients, including non-invasive blood pressure, pulse oximetry (SpO_2_) and electrocardiography, and baseline vital signs were obtained in addition to the tympanic membrane temperature: using a thermometer (FT 65 thermometer, Beurer^®^, Germany) pre-induction.

An epidural catheter (Perifix^®^, Braun, Germany) was inserted using complete aseptic technique through the midline approach and the loss of resistance was made using saline. Induction of general anaesthesia was accomplished using propofol (2 mg kg^-1^), fentanyl (1.0-2.0 µg kg^-1^), and rocuronium (0.6 mg kg^-1^). Intraoperatively, maintenance of anaesthesia was done using sevoflurane 2-3% in oxygen (inspiratory fraction 0.5 at a flow rate of 2-3 L min^-1^), and increments of rocuronium were administered. The patients were mechanically ventilated to keep end-tidal carbon dioxide between 30 and 35 mmHg. Through the epidural catheter, 20 mL of 0.125% levobupivacaine (Chirocaine^®^, Abbott) was injected followed by a continuous infusion of 0.125% levobupivacaine at 10 mL h^-1^. Tumescent fluid for liposuction was injected using 2 mm entry sites using a blunt-tipped infiltration needle connected by a large bore tube to an air-compressed pump.

Intraoperative hypothermia was minimized by several techniques, including the use of a heat and moisture exchanging filter placed between the endotracheal tube and the breathing circuit, warming all infused fluids, and the operating room temperature was adjusted to 22°C-24°C.

At 30 min before the termination of the procedure, participants were randomly allocated to 3 groups regarding the study drugs: group P (paracetamol group) (n = 40) who received intravenously 1 gram of paracetamol (Perfalgan, Bristol-Myers Squibb, Italy), group O (ondansetron group) (n = 40) who received intravenously 8 mg of ondansetron (Zofran, GlaxoSmithKline, Italy) and finally group S (saline group) (n = 40) who received normal saline. All drugs were infused over 15 min and in 100 mL volume.

At the end of the surgery, the residual muscle relaxant was reversed using 0.02 mg kg^-1^ atropine mixed with 0.04 mg kg^-1^ neostigmine, followed by endotracheal tube removal in the semi-sitting position after regaining consciousness. The extubation time (which is defined as the time from discontinuing anaesthetics till endotracheal tube removal), room temperature, total amount of drug consumption in the epidural anaesthesia, amount of tumescent fluid, blood loss, and total fluid consumption were recorded.

Patients were transferred to the recovery room and covered with a cotton blanket. The temperature of the post-op anaesthesia care unit was kept the same as that of the operating room by adjusting the air conditioner settings.

The body core temperature was recorded every 15 min for 60 min. Any episode of POS was recorded using the shivering score (SS): No shivering was scored as 0, piloerection or peripheral vasoconstriction was given a score of 1, muscular activity in only one muscle group was scored as 2, muscular activity in more than one muscle group was scored as 3 and shivering affecting the whole body was scored as 4.^13^ If POS grade was more than 3 for 15 min after administration of the test drug, meperidine 0.5 mg kg^-1^ was given intravenously as a rescue agent.

After 24 h of surgery, all patients were contacted to check their satisfaction with this technique, which was rated using 1-4 scales (1= poor; 2= fair; 3= good; 4= excellent).

The primary outcome was the incidence of POS in the first 60 min postoperatively as defined by a SS ≥3. The secondary outcome variables were to detect the time of the onset of POS by a SS of more than 3, in addition to comparing the score of the three groups and the total dose of meperidine administered.

### Statistical Analysis

The sample size of this trial was based on a pilot study with 10 participants to determine the incidence of POS after liposuction. It was reported in 80% of all patients. At least a 50% reduction in the incidence of POS in the first postoperative hour was accepted as clinically significant. Assuming an α error=0.05 with a power of 0.9, at least 33 patients per group were considered. To allow for patient withdrawal from the study, we decided to include 40 patients in each group.

Statistical Package for the Social Sciences (SPSS) software version 25.0 (SPSS Inc., Chicago, IL, USA) was used to analyze data. The Kolmogorov-Smirnov test was used to determine the normal distribution. The data were presented as the mean and standard deviation, and categorical data were expressed as the number of patients and incidence. The chi-square test or Fisher's exact test were used to compare categorical variables between the three groups, while a one-way analysis of variance was used to analyze continuous parametric variables, followed by post-hoc analysis (Tukey’s test) for intergroup comparisons. Moreover, the Kruskal-Wallis test was used to compare continuous non-parametric data variables, followed by post-hoc analysis (Mann-Whitney U test) for intergroup comparisons. *P* < 0.05 was considered statistically significant.

## Results

**Figure 1:**
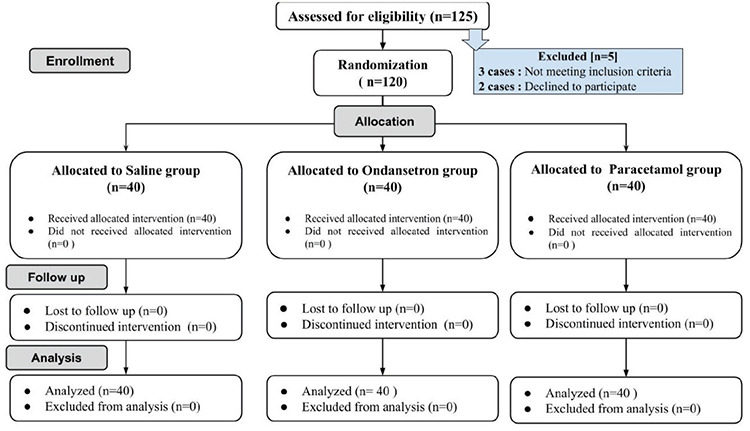
Consort flow diagram.

One hundred and twenty-five patients were eligible for enrollment; however, data from 120 patients (40 in each group) were collected and analyzed (Figure 1).

There were no significant differences between the three groups regarding demographic, anaesthetic, and operative data (Tables 1, 2). There were no clinically significant differences between the three groups concerning the amount of meperidine required to treat shivering or the response rate with value (83.3% in the *P* group, 66.7% in the O group, and 47.8% in the S group) (Table 3).

The incidence of POS (SS ≥3) was significantly lower in the P and O groups compared to the S group, with values 20% and 30% versus 65%, respectively (*P *< 0.001), while the onset of shivering was significantly later in the P and O groups compared to the S group with values of 24.38 ± 12.08 and 20.42 ± 10.33 versus 12.58 ± 6.83 respectively (*P *< 0.001). The need for additional antishivering treatment showed statistically significant differences between the studied groups, and was the most frequent in the S group when compared to the P and O groups with values 57.9% versus 15% and 22.5% respectively.

**Figure 2:**
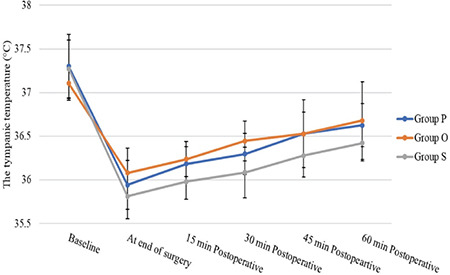
Patients’ core temperatures.

Furthermore, there was no significant difference in the frequency of postoperative complications recorded between the study groups (*P*=0.313) or postoperative patients’ core temperatures (Table 4) (Figure 2). One day after surgery, all patients were asked about their satisfaction with the shivering relief by using the study drug. Most patients were satisfied with the use of paracetamol or ondansetron (*P*=0.002) (Table 4), with no statistically significant difference between both study groups.

## Discussion

The present study was conducted to investigate the effects of the prophylactic use of either ondansetron or paracetamol given intraoperatively on the incidence and severity of POS in patients who had undergone mega liposuction. It was found that shivering was markedly reduced in the paracetamol and ondansetron groups (with no difference amongst these groups) compared with the saline (control) group. Moreover, it was established that using the study agents improved patient satisfaction postoperatively without affecting the occurrence of complications.

Paracetamol is an effective, safe and widely used analgesic agent with antipyretic properties that inhibits prostaglandin synthesis to reduce the hypothalamic temperature set point. It has a rapid onset of action about 15-20 min after the injection and declines after 4 h. Unlike other antishivering drugs, paracetamol does not cause adverse effects such as sedation, respiratory depression, constipation, or vomiting.^9^ Few studies have evaluated the feasibility of using paracetamol to treat postanaesthetic shivering.

The results of the current study agree with those of Kinjo et al.^14^, who found that the perioperative use of paracetamol could prevent severe POS in subjects who had undergone gynecological laparotomy. However, the study was conducted on a few patients compared to ours, and paracetamol was given after induction of anaesthesia and 4 h after the start of the surgery if the duration of surgery exceeded this time.

Moreover, a study conducted by Gholami and Hadavi^15^ also supports our study results, where prophylactic IV paracetamol was used during surgery on 110 pregnant women to prevent POS in cesarean delivery using general anaesthesia. The results showed a favorable response to prophylactic paracetamol regarding post-anaesthetic shivering; thus, it might replace opioids that have many side effects.

Data from 64 patients who underwent upper limb surgery under general anaesthesia in 2012 were collected by A. Khalili et al.^16^ studied the effects of intravenous paracetamol on POS and core and peripheral body temperature. Patients were divided into two groups: one group received 15 mg kg^-1^ and up to 1 g acetaminophen before induction of general anaesthesia, and the control group received normal saline. These results go along with our study results although both studies were conducted differently.

The study participants who underwent general anaesthesia for gynecological cancer surgery between 2012 and 2019 were given paracetamol to prevent POS, as demonstrated by Shirozu et al.^17^ in their retrospective study. These results are compatible with our study except that this study was retrospective, and the patients in each cohort were distributed unequally.^17^

Also, the results of the present study agree with those of Kashif et al.^18^, who evaluated the effect of pre-emptive intravenous acetaminophen on preventing POS in patients undergoing elective septoplasty under general anaesthesia. This study showed that pre-emptive use of 1 g of acetaminophen 20 min before completion of surgery decreases the incidence of POS.

Ondansetron, a specific 5-HT3 antagonist, has generated much interest because of its excellent pharmacological profile. It has a wide therapeutic index. It is usually prescribed to prevent and manage nausea and/or vomiting during the perioperative period. Currently, it is recommended for the prevention of POS at a dose of 4-8 mg.^19^

The exact mechanism of 5-HT3 antagonists in preventing postanaesthetic shivering has not been clarified, but it might be related to the inhibition of serotonin reuptake in the hypothalamus. Serotonin receptors also affect heat production and heat loss pathways, as well.^20^

The results of the present study are similar to a trial carried out by Mahoori et al.^21^, who had compared the efficacy of ondansetron and meperidine for treating shivering in 83 patients randomly divided into three groups: The first group was given 4 mg of IV ondansetron, the second group was given 8 mg of IV ondansetron, and the third group received 0.4 mg kg^-1^ of intravenous meperidine at the recovery room, and they found that 8 mg of IV ondansetron could control shivering and this is the dose of choice, especially in patients with POS in association of postoperative nausea and vomiting. These results were confirmed by Teymourian et al.^22^, where ondansetron was administered 10 min before the end of surgery to 40 patients for the prevention of post-anaesthesia shivering after elective craniotomy, and they found that ondansetron was of great value in preventing POS.

Also, in a study carried out by Abdollahi et al.^23^, who compared the efficacy of ondansetron and meperidine in preventing shivering after coronary artery bypass graft (CABG), they concluded that prophylactic administration of ondansetron 8 mg IV is equally effective as meperidine 0.4 mg kg^-1^ in the prevention of perioperative shivering in CABG patients.

An interesting meta-analysis of randomized controlled studies conducted by He et al.^20^ investigated the effectiveness and safety of ondansetron in preventing POS and concluded that treatment with ondansetron is both effective and safe as well as reducing POS.

Our results showed a significant reduction in incidence and severity of POS, and these results were against the results of a randomized clinical trial carried out by Browning et al.^24^ Who concluded that no significant difference between intravenous ondansetron 8 mg and placebo were given to parturient undergoing cesarean section under combined spinal-epidural anaesthesia. This may be explained by the criteria of these populations being all females, pregnant, and relatively young. There is evidence that POS in pregnancy and the peripartum period differs from thermoregulatory shivering seen in the non-pregnant population.^24^

Although Kelsaka et al.^25^ used 8 mg intravenous ondansetron in their study, a slightly higher percentage of patients in the ondansetron group had shivering (8% compared to 5.9% in our study). This may be due to their lower operating room temperature (21‑22°C). However, this has to be interpreted with caution since, contrary to expectation, a lower percentage of patients had to shiver in their control group compared to the control group in our trial (36% vs. 48.5%). The differences in the patient population in the two studies (non-obstetric versus obstetric patients) could also have accounted for the difference.

This study has some limitations. First, it was a single-center study, and the subjects were assessed for POS only for 60 min after the procedure. However, the incidence of POS can last up to 10 h.^4^ Second, we did not measure the plasma levels of paracetamol or ondansetron; however, this may not be practical. Further trials are needed to evaluate the late effects of paracetamol and ondansetron on POS and determine the optimal timing of administration for maximum benefit. Future studies should clarify the mechanism and optimal dose of paracetamol and ondansetron and determine which patient populations would most benefit from its use.

## Conclusion

In conclusion, in patients who have undergone liposuction under combined epidural and general anaesthesia, paracetamol is as effective as ondansetron when administrated at the end of surgery and can reduce the incidence and severity of POS.

## Figures and Tables

**Table 1:**
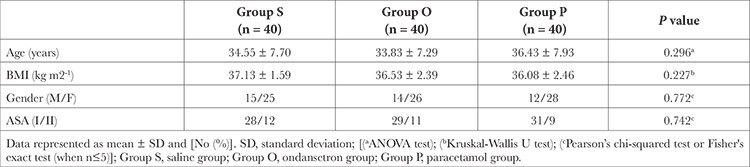
Demographic Data of Study Groups

**Table 2:**
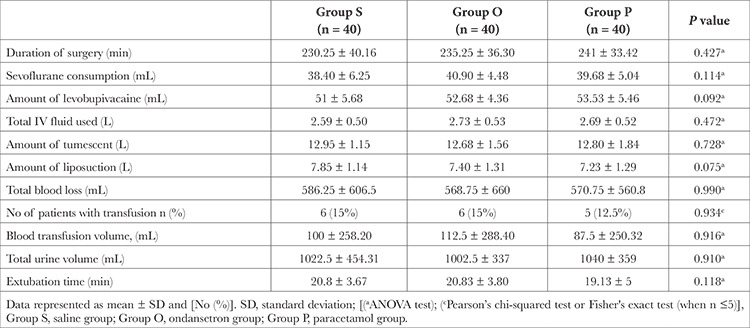
Anaesthetic and Operative Data of the Studied Groups

**Table 3:**
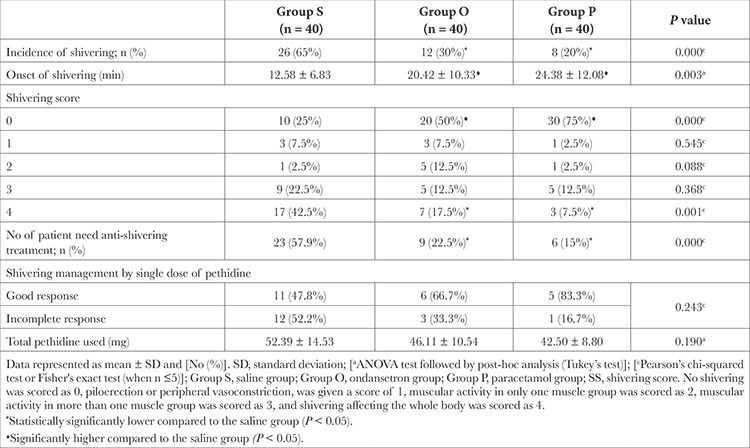
Incidence and Severity of Shivering

**Table 4:**
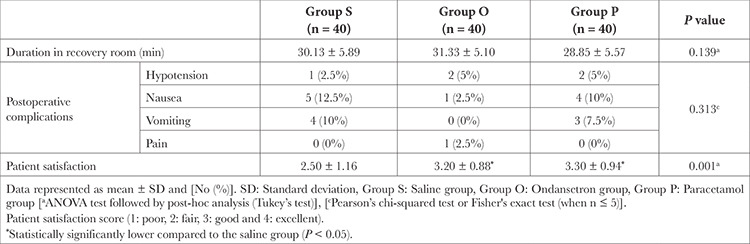
Postoperative Complications and Patient Satisfaction
